# Suppressing Cdk5 Activity by Luteolin Inhibits MPP^+^-Induced Apoptotic of Neuroblastoma through Erk/Drp1 and Fak/Akt/GSK3β Pathways

**DOI:** 10.3390/molecules26051307

**Published:** 2021-02-28

**Authors:** Ratchaneekorn Reudhabibadh, Thunwa Binlateh, Pennapa Chonpathompikunlert, Nongyao Nonpanya, Peerada Prommeenate, Pithi Chanvorachote, Pilaiwanwadee Hutamekalin

**Affiliations:** 1 Division of Health and Applied Sciences, Faculty of Science, Prince of Songkla University, Hat Yai, Songkhla 90110, Thailand; ratchaneekorn.sru@gmail.com (R.R.); thunwa.bin@gmail.com (T.B.); 2 Expert Centre of Innovative Health Food (InnoFood), Thailand Institute of Scientific and Technological Research (TISTR), Khlong Luang, Pathum Thani 12120, Thailand; pennapa@tistr.or.th; 3 Cell-Based Drug and Health Products Development Research Unit, Faculty of Pharmaceutical Sciences, Chulalongkorn University, Bangkok 10330, Thailand; nongyaononpanya@gmail.com (N.N.); pithi.c@chula.ac.th (P.C.); 4 Biochemical Engineering and Systems Biology Research Group, National Center for Genetic Engineering and Biotechnology, National Science and Technology Development Agency at King Mongkut’s University of Technology Thonburi, Bangkok 10150, Thailand; pprommeenate@gmail.com

**Keywords:** 1-methyl-4-phenylpyridinium ion, apoptosis, Cdk5, luteolin, oxidative stress, Parkinson’s disease

## Abstract

Parkinson’s disease (PD) is characterized by the progressive degeneration of dopaminergic neurons. The cause of PD is still unclear. Oxidative stress and mitochondrial dysfunction have been linked to the development of PD. Luteolin, a non-toxic flavonoid, has become interested in an alternative medicine, according to its effects on anti-oxidative stress and anti-apoptosis, although the underlying mechanism of luteolin on PD has not been fully elucidated. This study aims to investigate whether luteolin prevents neurotoxicity induction by 1-methyl-4-phenylpyridinium iodide (MPP^+^), a neurotoxin in neuroblastoma SH-SY5Y cells. The results reveal that luteolin significantly improved cell viability and reduced apoptosis in MPP^+^-treated cells. Increasing lipid peroxidation and superoxide anion (O_2_^−^), including mitochondrial membrane potential (Δψm) disruption, is ameliorated by luteolin treatment. In addition, luteolin attenuated MPP^+^-induced neurite damage via GAP43 and synapsin-1. Furthermore, Cdk5 is found to be overactivated and correlated with elevation of cleaved caspase-3 activity in MPP^+^-exposed cells, while phosphorylation of Erk1/2, Drp1, Fak, Akt and GSK3β are inhibited. In contrast, luteolin attenuated Cdk5 overactivation and supported phosphorylated level of Erk1/2, Drp1, Fak, Akt and GSK3β with reducing in cleaved caspase-3 activity. Results indicate that luteolin exerts neuroprotective effects via Cdk5-mediated Erk1/2/Drp1 and Fak/Akt/GSK3β pathways, possibly representing a potential preventive agent for neuronal disorder.

## 1. Introduction

Parkinson’s disease (PD) is a progressive neurodegenerative disease characterized by the abnormal aggregation of α-synuclein protein and death of dopaminergic neurons in the substantia nigra pars compacta [1,2]. At present, the pathogenesis of PD is still not fully understood. Nonetheless, mitochondrial dysfunction and oxidative stress have been reported as the important pathogenic factors [3]. An active metabolite of 1-methyl-4-phenyl-1,2,6-tetrahydropyridine (MPTP), 1-methyl-4-phenylpyridinium ion (MPP^+^), is highly toxic to dopaminergic neurons, and has successfully induced Parkinson-like syndromes in an in vitro model [4]. MPP^+^ induces the production of massive reactive oxygen species (ROS) in mitochondria through oxidative phosphorylation, which leads to neuronal damage [5]. The overproduction of ROS impairs cell membrane structure, and affects its biological compositions such as lipids, proteins and DNAs, which then triggers neuronal apoptosis [6]. Moreover, the cytoskeleton is also disrupted by excessive ROS, resulting in synaptic dysfunction [7]. It has been reported that MPP^+^ markedly decreased synaptic plasticity through synapsin-1 and growth associated protein 43 (GAP43), which is correlated with cell death [8].

During the PD symptom development, ROS interact with the protein involved in the pathology of PD, and contribute to neuronal cell death [9]. Cyclin-dependent kinase-5 (Cdk5) is reported as an important contributor on PD pathology through ROS interaction [10]. Cdk5 is a serine/threonine kinase that is activated upon association with its activator p35 [11]. It was found that overexpression of Cdk5 caused by ROS has a key role in neuronal apoptosis through the phosphorylation of its downstream substrates [10]. A previous study reported that an elevated level of Cdk5 suppresses the function of extracellular signal-regulated kinases 1/2 (Erk1/2), leading to cell death [12]. Erk1/2 is a protein-serine/threonine kinase that participates in the Ras-Raf-MEK-ERK signal transduction cascade, and plays a central role in regulating mitochondrial function [13]. Furthermore, Erk1/2 has reportedly been associated with survival-death decision via phosphorylation of dynamin-related protein 1 (Drp1) [14]. Drp1 is a cytosolic protein that regulates mitochondrial dynamics. Phosphorylated Drp1 has been known to promote mitochondrial fission and improve mitochondrial respiratory function [15]. Inhibition of Drp1 triggers apoptosis and links to aging and neurodegenerative development [16,17]. Focal adhesion kinase (Fak) is another downstream target of Cdk5. Fak is a cytoplasmic protein tyrosine kinase that plays a crucial role in cell survival and synaptic function via phosphorylation of protein kinase B (Akt) [18,19]. A previous study demonstrated that the knockdown and suppression of Fak are able to induce apoptosis by inhibiting Akt function [20]. In addition, the phosphorylation of Akt by Fak brings on an increase in the phosphorylation of glycogen synthesis kinase 3β (GSK3β), which demonstrated the suppression of apoptosis by inhibiting proapoptotic Bax [21,22].

Many preclinical studies suggest that consumption of natural flavonoids plays an important role in neuroprotection by modulating cellular signaling pathways [23,24]. Luteolin (3′,4′,5,7-tetrahydroxyflavone), a common flavonoid found in celery, green pepper and other herbs, is reported to possess multiple pharmacological properties, including anticancer, anti-oxidant and anti-apoptosis properties [24,25]. Luteolin has previously been used in pharmacological studies against ROS-involved neuronal degeneration through Akt and Erk1/2 pathways [26,27]. In addition, luteolin could ameliorate the neuroplasticity dysfunctions by upregulating the expression of GAP43 [28]. Although luteolin was reported for neuroprotection effect, the underlying mechanism needs further elucidation. However, an increasing number of studies have proven that luteolin could possess neuroprotective properties through suppressing the overexpression of Bax, the activation of anti-apoptotic Bcl-2 protein and the scavenging of ROS [25,26].

Currently, various pharmacological treatments were used in PD patients, although some of these have significant adverse effects, and do not retard the degeneration of neu-rons. In this study, we therefore explored the effects of luteolin on MPP^+^-induced apoptosis in human SH-SY5Y neuroblastoma cells.

## 2. Results

### 2.1. Luteolin Prevented MPP^+^-Induced Neurotoxicity in SH-SY5Y Cells

**Figure 1:**
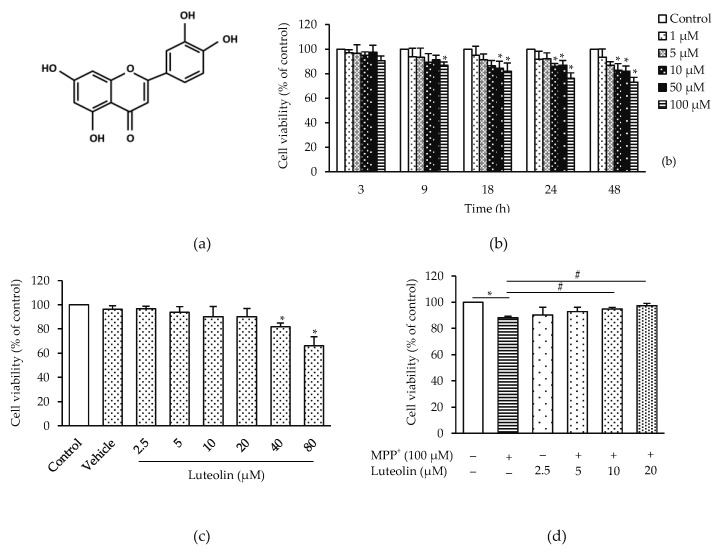
Luteolin prevented 1-methyl-4-phenylpyridinium iodide (MPP+)-induced neurotoxicity in SH-SH5Y cells. (**a**) Chemical structure of luteolin. (**b**) The cells were treated with various concentrations of MPP^+^ (0–100 µM) for 3, 9, 18 and 24 h. (**c**) The cells were treated with various concentrations of luteolin (0–80 µM) for 24 h. (**d**) The cells were pre-treated with luteolin (2.5–20 µM) for 1 h followed by 100 µM MPP^+^ for 24 h. Cell viability was measured by 0.5 mg/mL of 3-(4,5-dimethylthiazol-2-yl)-2,5-diphenyl tetrazolium bromide (MTT) assay. Results are shown as the mean ± SD for triplicate independent experiments. Differences are statistically significant at * *p* < 0.05 versus the control group, and ^#^ *p* < 0.05 versus the MPP^+^ group.

To investigate the neuroprotective effect of luteolin, MPP^+^ was applied to trigger cytotoxicity in SH-SY5Y cells. Cells treated with MPP^+^ (1–100 µM) for 3–24 h showed significantly reduced cell viability in concentration, in a time-dependent manner (Figure 1b). MPP^+^ at the concentration of 100 µM was chosen for following experiments, according to its significantly reduced cell viability by 25.39% at 24 h. The toxicity of luteolin at its different concentration (2.5–80 µM) on cell viability was further investigated. Treatments for 24 h with 2.5–20 µM luteolin showed no different effects, while 40–80 µM luteolin presented significantly different cell viability reduction, compared to the control group (Figure 1c). To further investigate whether luteolin could alleviate MPP^+^-induced cytotoxicity, cells were pre-treated with 2.5–20 µM luteolin for 1 h, followed by the incubation with 100 µM MPP^+^ for 24 h. As shown in Figure 1d, MPP^+^ induced a significant decrease in cell viability compared to the control group, while pre-treatment with 10 µM and 20 µM luteolin prevented MPP^+^-induced cytotoxicity by restoring cell viability. Twenty µM luteolin revealed the highest effects, and therefore this concentration was chosen for further experiments. 

### 2.2. Luteolin Protected MPP^+^-Induced Apoptosis in SH-SY5Y Cells 

**Figure 2:**
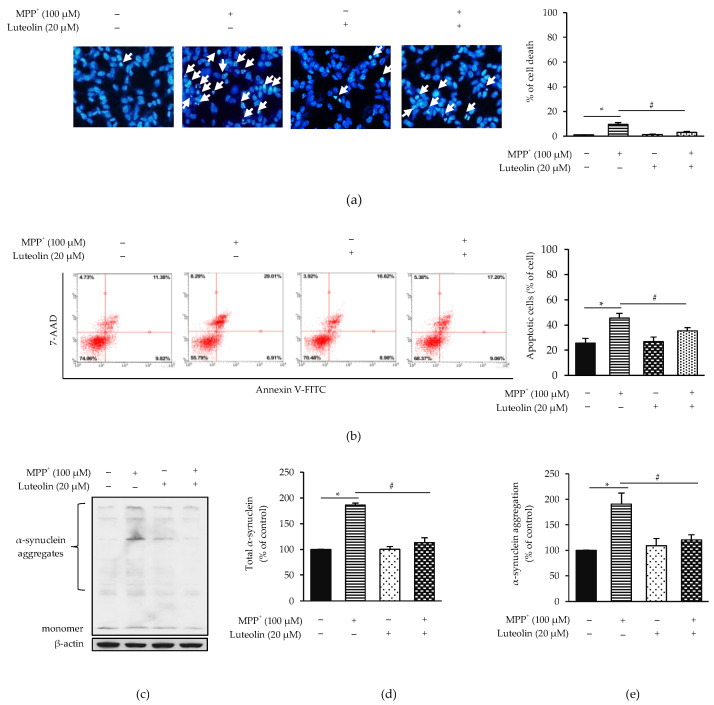

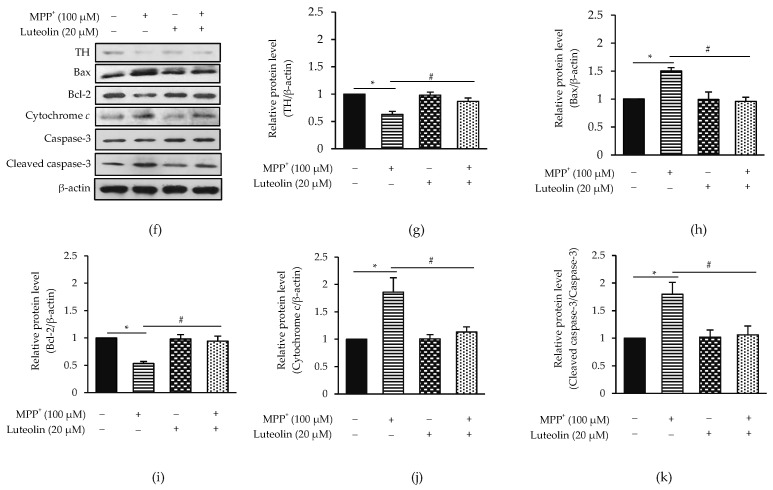
Luteolin alleviate MPP^+^-induced apoptosis in SH-SY5Y cells. Cells were pre-treated with 20 µM luteolin for 1 h, followed by 100 µM MPP^+^ for 24 h. After 24 h, apoptotic cells were evaluated by Hoechst 33342 staining, Annexin V-FITC/7-ADD staining and western blotting. (**a**) Stained cells with Hoechst 33342. Nuclear condensation and nuclear fragmentation were observed under fluorescence microscope (20×). Bar graph represented percentage of apoptotic nuclei. (**b**) Stained cells with Annexin V-FITC/7-ADD and flow cytometry was then applied for identifying apoptotic cells. Bar graph represents the percentage of apoptotic cells. (**c**,**f**) Expressions of α-synuclein, TH, Bax, Bcl-2, cytochrome c, capase-3 and cleaved caspase-3 were measured by western blot analysis. Quantification of (**d**,**e**) α-synuclein (**g**) TH, (**h**) Bax, (**i**) Bcl-2, (**j**) cytochrome c and (**k**) cleaved caspase-3/capase-3 was shown in the bar graph. Data were normalized using β-actin as control. Results are shown as mean ± SD for triplicated independent experiments. Differences are statistically significant at * *p* < 0.05 versus the control group and ^#^ *p* < 0.05 versus the MPP^+^ group.

Since apoptosis is considered as one of the MPP^+^-induced neuronal injury pathogenesis, luteolin attenuated MPP^+^-triggered neurotoxicity activity via inhibiting apoptosis was investigated. Hoechst 33342 staining and flow cytometry analysis were performed in pre-treatment cells with luteolin, followed by MPP^+^. Pre-treatment with luteolin significantly decreased the number of apoptotic cells, in comparison to MPP^+^-treated cells as the control group (Figure 2a). Likewise, flow cytometric data showed that luteolin significantly reduced the percentage of apoptotic cell death induced by MPP^+^ in the same way as the Hoechst staining results (Figure 2b). In addition, the neuroprotective effects of luteolin on MPP^+^-induced apoptosis were confirmed by western blot analysis using the neuropathological hallmark protein of PD, such as dopaminergic neuronal protein marker and apoptotic protein, including α-synuclein, TH, Bax, Bcl-2, cytochrome *c*, caspase-3 and cleaved caspase-3. Results showed that MPP^+^ significantly induced α-synuclein aggregation, expression of Bax, cytochrome *c* and cleaved caspase-3/caspase-3, while the TH level (a rate-limiting enzyme mainly expressed in dopaminergic neurons) and Bcl-2 expression were decreased, compared to the control group. On the other hand, the pre-treatment of luteolin reversed the expression of these proteins back to normal levels, with no statistical difference to the control group (Figure 2c–k). These findings suggested that luteolin protected MPP^+^-induced neurotoxicity by inhibiting apoptotic proteins.

### 2.3. Luteolin Ameliorated MPP^+^-Reduced Synaptic Communication via GAP43 and Synapsin-1

**Figure 3:**
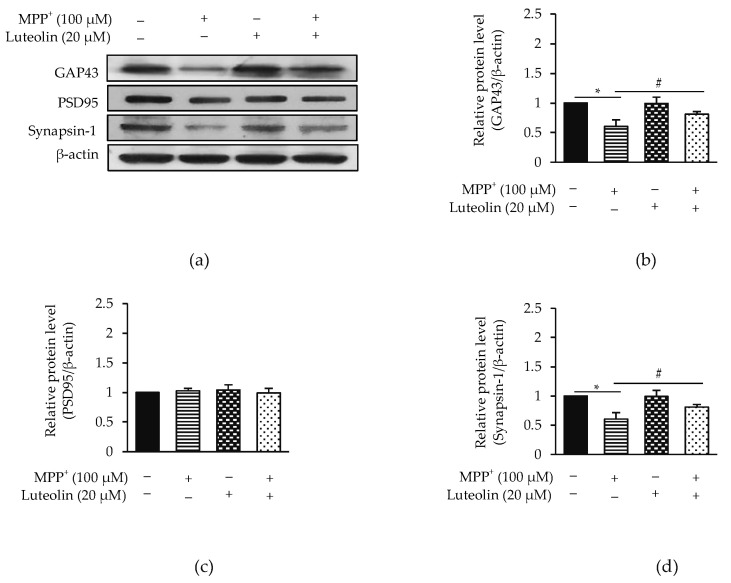
Luteolin prevented MPP^+^-suppressed synaptic communication in SH-SY5Y cells. Cells were pre-treated with 20 µM luteolin for 1 h followed by treatment with 100 µM MPP^+^ for 24 h. (**a**) Expressions of GAP43, PSD95 and synapsin-1 were detected by western blot analysis. Bar graph represents (**b**) GAP43, (**c**) PSD95 and (**d**) synapsin-1 expression levels. Data were normalized using β-actin as control. Results are shown as the mean ± SD for triplicated independent experiments. Differences are statistically significant at * *p* < 0.05 versus the control group, and ^#^ *p* < 0.05 versus the MPP^+^ group.

The previous study revealed that synaptic loss is an early event in neurodegenerative diseases [8]. An investigation on the cellular protecting effect of luteolin from MPP^+^-decreased neuronal synaptic plasticity was then performed. Expressions of GAP43, PSD95 and synapsin-1 were evaluated (Figure 3a). Cells treated with MPP^+^ were significantly decreased in GAP43 and synapsin-1 without any effect on PSD95 expression levels. The adding of luteolin revealed the reverse MPP^+^ effects on GAP43 and synapsin-1 reduction (Figure 3b–d). These findings demonstrated that luteolin could prevent MPP^+^-induced synaptic loss through activities of GAP43 and synapsin-1.

### 2.4. Luteolin Inhibited the Accumulation of Intracellular ROS Induced by MPP^+^

**Figure 4:**
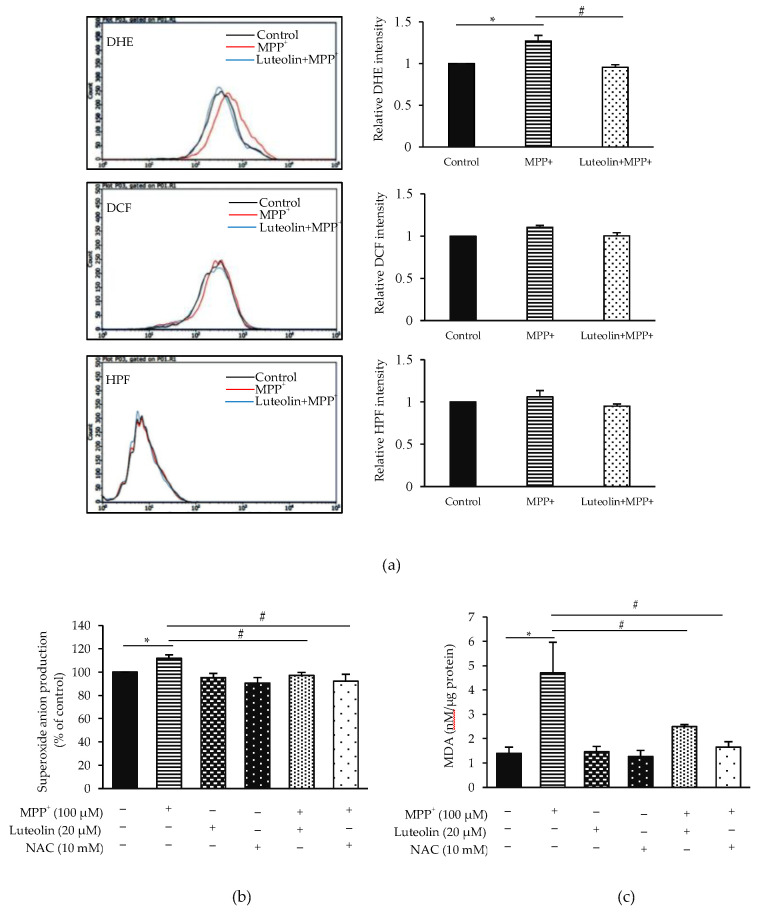
Luteolin protected MPP^+^-induced intracellular reactive oxygen species (ROS) and malondialdehyde (MDA) generation in SH-SY5Y cells. (**a**) The cells treated with 100 µM MPP^+^ for 24 h in the presence or absence of 20 µM luteolin were incubated with specific ROS fluorescent probes, including DHE, DCF or HPF for 3 h. Fluorescence intensity of dihydroethidium (DHE), 2′,7′-dichlorofluorescein (DCF) or hydroxyphenyl fluorescein (HPF) was analyzed by flow cytometry. Cells were pre-incubated with 20 µM luteolin and 10 mM NAC for 1 h, followed by 100 µM MPP^+^. Levels of (**b**) O_2_^−^ and (**c**) MDA were measured by xanthine/xanthine oxidase systems and thiobarbituric acid reactive substances (TBARS) assay, respectively. Results presented as the mean ± SD for triplicated independent experiments. Differences are statistically significant at * *p* < 0.05 versus the control group and ^#^ *p* < 0.05 versus the MPP^+^ group. DHE: dihydroethidium, DCF: 2′,7′-dichlorofluorescein, HPF: hydroxyphenyl fluorescein and NAC: N-acetyl cysteine.

To determine the scavenging effects of luteolin on MPP^+^-induced ROS generation, levels of superoxide anion (O_2_^−^), hydrogen peroxide (H_2_O_2_) and hydroxyl radical (OH^−^) were assessed by dihydroethidium (DHE), 2′,7′-dichlorofluorescein (DCF) and hydroxyphenyl fluorescein (HPF), respectively. As shown in Figure 4a, relative fluorescent intensity showed that treating cells with MPP^+^ significantly induced O_2_^−^ generation, while there was no detectable effect on H_2_O_2_ and OH^−^ compared to the control group. In contrast, pre-treatment with luteolin significantly attenuated MPP^+^-induced O_2_^−^ elevation, compared to the MPP^+^ treated group. Next, ROS scavenger NAC was added into this study in order to confirm the antioxidant effect of luteolin on MPP^+^-induced oxidative stress. Figure 4b,c showed that exposure to MPP^+^ significantly increased the malondialdehyde (MDA), a biomarker of oxidative damage and O_2_^−^ levels, compared to the control group, while administration of luteolin and NAC significantly attenuated these effects. Results suggested that luteolin inhibited MPP^+^-induced ROS production.

### 2.5. Luteolin Inhibited the Reduction of Mitochondrial Membrane Potential (Δψm) Induced by MPP^+^

**Figure 5:**
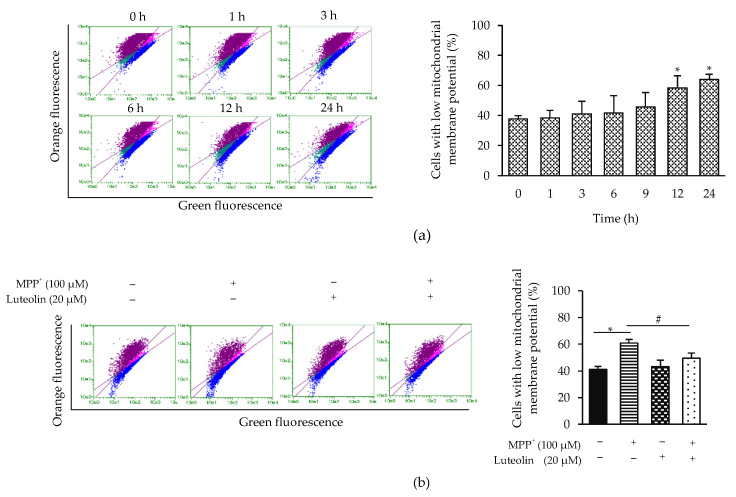
Luteolin protected MPP^+^-induced Δψm loss in SH-SY5Y cells. (**a**) Cells were incubated with 100 µM MPP^+^ for 0, 1, 3, 6, 9, 12 and 24 h. (**b**) Cells were pre-treated with 20 µM luteolin for 1 h, followed by 100 µM for 12 h. Cells were then incubated with JC-1 dye, and Δψm was determined by flow cytometry. Results presented as the mean ± SD for triplicated independent experiments. Differences are statistically significant, at * *p* < 0.05 versus the control group and ^#^ *p* < 0.05 versus the MPP^+^ group.

Reduction of Δψm has been shown to participate in apoptosis [29]. Effects of luteolin on MPP^+^-induced Δψm loss were then investigated. JC-1 dye was used for Δψm detection. Exposure of MPP^+^ increased cell percentage with low Δψm in a time-dependent manner, indicating the mitochondria damage by MPP^+^ (Figure 5a). Importantly, pre-treatment with luteolin significantly attenuated Δψm disruption by MPP^+^ stimulation (Figure 5b). 

### 2.6. Luteolin Ameliorated MPP^+^-Induced Apoptosis via Cdk5

**Figure 6:**
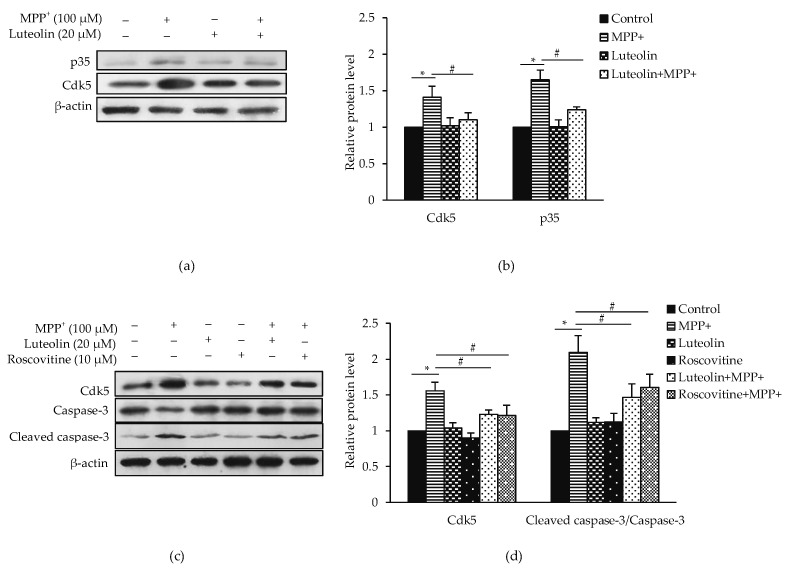
Luteolin ameliorated MPP^+^-induced Cdk5 hyperactivity in SH-SY5Y cells. Cells were pre-treated with 20 µM luteolin for 1 h, followed by treatment with 100 µM MPP^+^ for 24 h. (**a**) Expressions of Cdk5 and p35 were analyzed by western blotting. (**b**) Quantification of Cdk5 and p35 was shown in the bar graph. Cells were pre-treated with 20 µM luteolin and 10 µM roscovitine for 1 h, followed by treatment with 100 µM MPP^+^ for 24 h. (**c**) Expressions of Cdk5 and cleaved caspase-3/caspase-3 were analyzed by western blotting. (**d**) Quantification of Cdk5 and cleaved caspase-3/caspase-3 was shown in the bar graph. Data were normalized using β-actin as control. Results presented as the mean ± SD for triplicated independent experiments. Differences are statistically significant at * *p* < 0.05 versus the control group, and ^#^ *p* < 0.05 versus the MPP^+^ group.

Cdk5/p35 is a key regulator dopaminergic neurons degeneration in PD and MPP^+^ neurotoxicity induction [12]. To evaluate whether the neuroprotective effects of luteolin on MPP^+^-induced cell death are involved in the Cdk5/p35 signaling pathway, the expression of Cdk5 and p35 was evaluated (Figure 6a). Expression of Cdk5 and p35 was significantly increased in treated cells with MPP^+^, while the expressions were significantly reduced in pre-treatment with luteolin (Figure 6b). To investigate the role of Cdk5 in MPP^+^-induced cell death, a Cdk inhibitor, roscovitine, was applied and used as a positive control. Luteolin and roscovitine significantly inhibited expressions of Cdk5 and cleaved caspase-3/caspase-3, indicating that the protective mechanism of luteolin on MPP^+^-induced apoptosis perhaps relates to Cdk5 activity (Figure 6c,d).

### 2.7. Luteolin Inhibited MPP^+^-Reduced Erk1/2 and Drp1 via Cdk5

**Figure 7:**
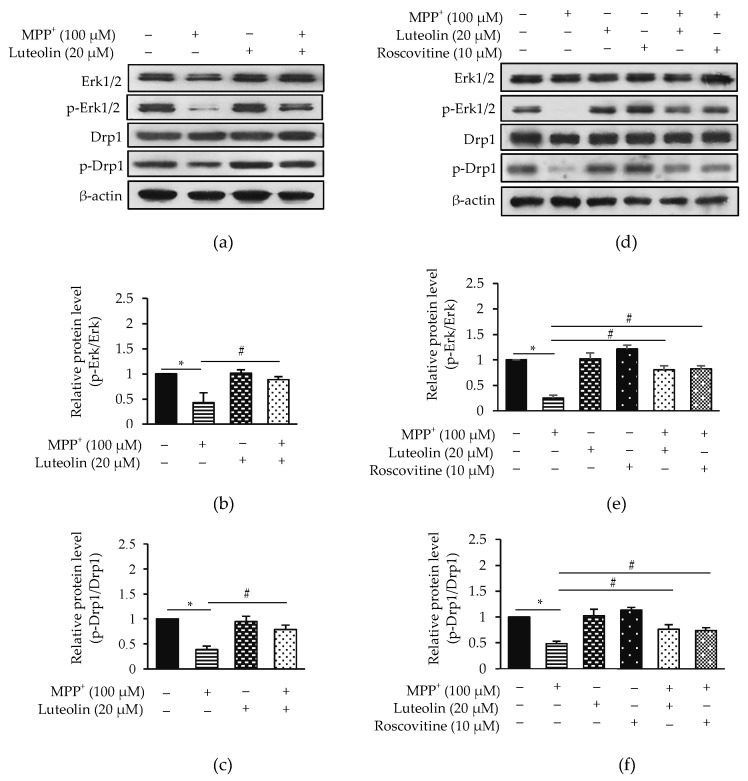
Luteolin protected MPP^+^-suppressed Erk1/2 and Drp1 in SH-SY5Y cells. Cells were pre-treated with 20 µM luteolin for 1 h, followed by treatment with 100 µM MPP^+^ for 24 h. (**a**) Expressions of Erk, p-Erk, Drp1 and p-Drp1 were analyzed by western blot analysis. Quantification of (**b**) p-Erk/Erk and (**c**) p-Drp1/Drp1 was shown in the bar graph. Cells were pre-treated with 20 µM luteolin and 10 µM roscovitine for 1 h, followed by treatment with 100 µM MPP^+^ for 24 h. (**d**) Expressions of Erk, p-Erk, Drp1 and p-Drp1 were analyzed by western blotting. Quantification of (**e**) p-Erk/Erk and (**f**) p-Drp1/Drp1 was shown in the bar graph. Data were normalized using β-actin as control. Results presented as the mean ± SD for triplicated independent experiments. Differences are statistically significant at * *p* < 0.05 versus the control group, and ^#^ *p* < 0.05 versus the MPP^+^ group.

Previous studies have reported that the expression of Erk1/2 and Drp1 is correlated with cell survival and apoptosis [14]. To investigate the mechanisms of luteolin in cell protection from MPP^+^-induced apoptosis, protein expressions of Erk1/2 and Drp1 were evaluated by western blot analysis (Figure 7a). The expression of p-Erk/Erk and p-Drp1/Drp1 was significantly reduced in treated cells with MPP^+^, while pre-treatment cells with luteolin prior to exposure to MPP^+^ were increased significantly (Figure 7b,c). To affirm whether luteolin provided a protective effect via Cdk5, a Cdk inhibitor roscovitine was applied. Roscovitine significantly induced the expression of p-Erk/Erk and p-Drp1/Drp1 compared to MPP^+^-treated cells (Figure 7d–f). Results indicated that protective mechanism of luteolin on MPP^+^-induced apoptosis may relate to inhibiting Cdk5 and up-regulating Erk1/2 and Drp1.

### 2.8. Luteolin Suppressed MPP^+^-Inhibited Fak/Akt/GSK3β through Cdk5

**Figure 8:**
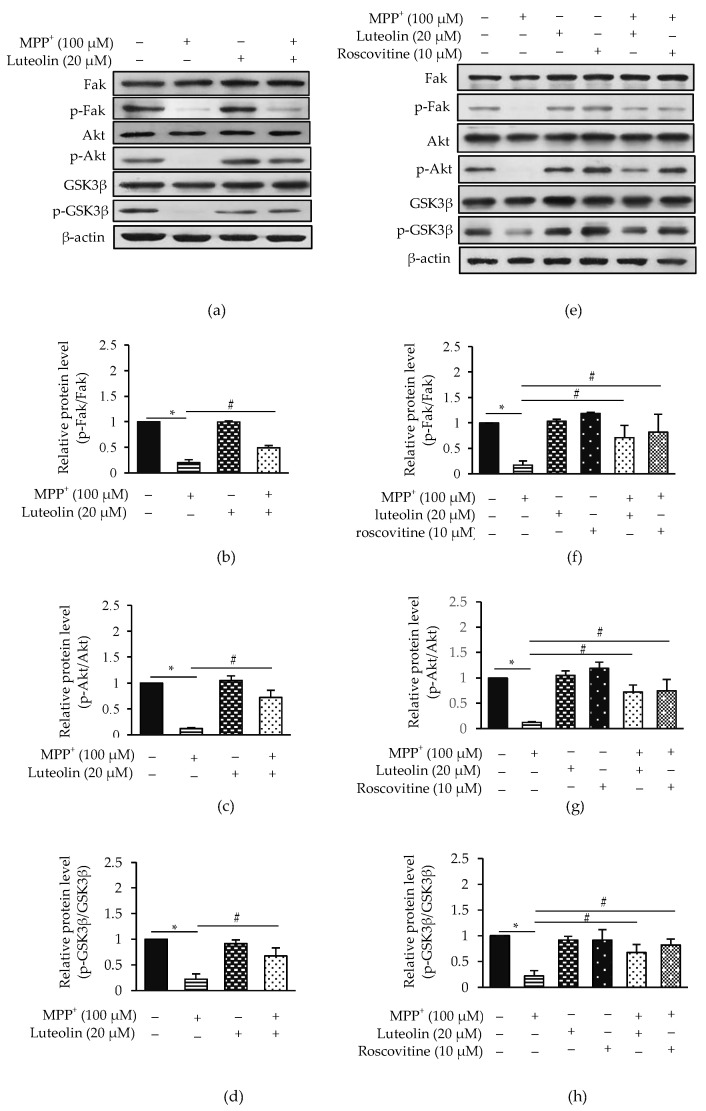
Luteolin ameliorated MPP^+^-suppressed Fak/Akt/GSK3β in SH-SY5Y cells. Cells were pre-treated with 20 µM luteolin for 1 h, followed by treatment with 100 µM MPP^+^ for 24 h. (**a**) Expressions of Fak, p-Fak, Akt, p-Akt GSK3β and p-GSK3β were analyzed using western blotting. Quantification of (**b**) p-Fak/Fak, (**c**) p-Akt/Akt and (**d**) p-GSK3β/GSK3β was shown in a bar graph. Cells were pre-treated with 20 µM luteolin and 10 µM roscovitine for 1 h, followed by treatment with 100 µM MPP^+^ for 24 h. (**e**) Expressions of Fak, p-Fak, Akt, p-Akt GSK3β and p-GSK3β were analyzed by western blotting. Quantification of (**f**) p-Fak/Fak, (**g**) p-Akt/Akt and (**h**) p-GSK3β/GSK3β was shown in the bar graph. Data were normalized using β-actin as a control. Results presented as the mean ± SD for triplicated independent experiments. Differences are statistically significant at * *p* < 0.05 versus the control group, and ^#^ *p* < 0.05 versus the MPP^+^ group.

It has been reported that Fak, Akt and GSK3β are essential for cell survival [18,21]. To further investigate the protective mechanism of luteolin on MPP^+^-induced apoptosis, expressions of Fak, Akt and GSK3β were determined (Figure 8a). Results showed that MPP^+^ significantly reduced the expressions of p-Fak/Fak, p-Akt/Akt and p-GSK3β/GSK3β while pre-treatment with luteolin ameliorated these changes (Figure 8b–d). To further confirm the role of Cdk5 on Fak, Akt and GSK3β activities in MPP^+^-induced cell death, a Cdk inhibitor roscovitine was applied, as in the previous experiment. Roscovitine significantly reversed the expression of p-Fak/Fak, p-Akt/Akt and p-GSK3β/GSK3β when compared to the MPP^+^-treated group, indicating the down-regulation of Fak, Akt and GSK3β (Figure 8e–h). These results suggested a potent neuroprotective effect against MPP^+^-induced cell death via the Fak/Akt/GSK3β pathway of luteolin.

## 3. Discussion

Parkinson’s disease (PD) is a neurodegenerative disease triggered by multiple pathogenic factors. Several studies have indicated that ROS is intimately linked with the pathology of PD [9,30]. However, the mechanisms of ROS involved in signaling molecules of PD conditions are unknown. MPP^+^ is a neurotoxin which is recognized as a valuable tool to mimic dopaminergic degeneration [31]. Many experiments with neurons have shown that the toxicity of MPP^+^ was observed at different concentrations. To test the suitability of our model system, cells were exposed to MPP^+^ in the range of 1–100 M for 3–24 h. The results showed that MPP^+^ exhibited a dose-time dependent cytotoxicity. A previous study has reported that luteolin has multiple bioactivities and neuroprotective effects [24]. The protective effect of luteolin on MPP^+^-induced neurotoxicity was further examined. Pre-treatment with 20 µM luteolin markedly reversed the effect of 100 µM MPP^+^. The data was consistent with a previous study, in that 20 µM luteolin can ameliorate cytotoxicity induced by 6-OHDA [32]. Therefore, 20 µM luteolin was chosen as an optimal concentration for further experiments.

Loss of dopaminergic neuron is a major pathology found in PD [2]. Post-mortem studies reveal the involvement of mitochondrial-dependent apoptotic characteristics in the loss of dopaminergic neuron, including decreased Bcl-2 and increased Bax, cytochrome *c* and caspase-3 [33]. A previous study has reported that MPP^+^ is involved in the cell apoptosis mechanism [29]. In this present study, MPP^+^ generated apoptosis in SH-SY5Y cells by activating mitochondrial-dependent apoptosis as detected by an increase of Bax, cytochrome c and caspase-3, and a decrease of Bcl-2 protein expressions. Luteolin could prevent the MPP^+^-induced apoptosis by increasing the expression of the Bcl-2 protein. These results are consistent with previous findings, in that luteolin could protect H_2_O_2_-induced cell apoptosis through the Bcl-2 pathway [25]. The finding demonstrated that luteolin possessed anti-apoptotic property in MPP^+^-induced SH-SY5Y apoptotic cell death. Further evidence for synaptic loss is an early event in neurological diseases, and therefore the regeneration of neurite networks is an interesting strategy for disorder treatment [8]. GAP43 and synapsin-1 are crucial in neuronal development and synaptic physiology [34,35]. There is suggested evidence that synaptic disruption is first affected in PD, while MPP^+^ is able to induce synaptic plasticity and cell viability reduction [36,37]. Data in this study agree with the finding that MPP^+^ induced synaptic plasticity degeneration by decreasing GAP43 and synapsin-1, while luteolin could reduce cell loss and improve neuronal synaptic plasticity [28,38]. Results suggested that luteolin exhibited neuroprotective property against MPP^+^-induced apoptosis and synaptic damage in SH-SY5Y cells.

ROS is reported as a key factor in PD pathogenesis [30]. An increase of ROS causes over-production of MDA, which is a marker of oxidative stress [39]. MPP^+^ has been involved in neuronal cell death by inducing ROS production which then disrupt Δψm and further resulting in neuronal cell death [12,29]. These results also supported the previous findings that MPP^+^ induced Δψm loss and elevation in O_2_^−^ and MDA production [40]. In this experiment, however, pre-treatment with luteolin reduces MPP^+^-induced Δψm loss, while also decreasing O_2_^−^ and MDA generation. Previous studies also revealed the important role of luteolin in protecting biological systems from oxidative stress [41]. Luteolin improved superoxide dismutase activity, which consequently prevents ROS generation. Moreover, luteolin could promote the levels of glutathione peroxidase and glutathione activities, which were found as important anti-oxidant enzymes to decompose OH^−^ mediated lipid peroxidation [41]. Data in this study implicated that the inhibition of Δψm loss, O_2_^−^ and MDA are involved with protective effects of luteolin against MPP^+^-induced toxicity. 

Although ROS primarily act to trigger oxidative stress, accumulating studies suggested that Cdk5 hyperactivation is another key mechanism of ROS-mediated neuronal cell death [10]. Hence, to unravel the role of Cdk5 in ROS-mediated neuronal cell death, we applied a Cdk inhibitor, roscovitine, in this study. Results agree with previous find-ings that MPP^+^ induced neuronal cell apoptosis via Cdk5 and p35 [12]. Pre-treatment with luteolin diminished MPP^+^-induced apoptosis by suppressing Cdk5 and p35 expression. Inhibition of Cdk5/p35 may be due to the anti-oxidative effect of luteolin on MPP^+^-induced oxidative stress [25].

It is well accepted that Cdk5 is not only associated with cytoskeletal phosphorylation, but also involved in modulating multiple kinase activities in the contribution of neurodegenerations [10]. Cdk5 is reported to control Erk1/2, which is linked to mitochondrial fission through phosphorylation of Drp1 [14,42]. A previous study revealed that MPP^+^ induced SH-SY5Y cell apoptosis through increasing Cdk5 simultaneously with the reduction of Erk1/2 activity, which correlated with decreasing mitochondrial fission and Drp1 gene expression in neurotoxin rotenone-treated primary cortical neurons [12,43]. However, the link between Cdk5 and apoptosis through Erk1/2 and Drp1 remains obscure. In this study, the experimental data supported that MPP^+^ could reduce Erk1/2 and Drp1 expression, while pre-treatment of the cells with luteolin and roscovitine could increase Erk1/2 and Drp1 expression. These findings are consistent with previous reports that luteolin helps in apoptosis prevention through increased Erk1/2 and Drp1 expression. Therefore, these results indicated that luteolin improved the Erk/Drp1-dependent survival pathway via suppressing Cdk5. 

Another study has indicated that Cdk5 is also involved in the phosphorylation of Fak^ser732^, which is responsible for cell proliferation, migration and survival [44,45]. Fak mediates cell survival via the cascade of Akt phosphorylation, followed by increasing GSK3β phosphorylation [46,47]. Phosphorylation of GSK3β ameliorated apoptosis by facilitating Bcl-2 and inhibiting p53, which led to decreasing Bax [22]. These results showed that MPP^+^ markedly induced cell apoptosis via the inhibition of phosphorylated Akt and GSK3β protein expression, which are the downstream targets of Fak. However, pre-treatment with luteolin could reverse the expression of these proteins. Currently, results supported that luteolin markedly upregulated p-GSK3β and Bcl-2 protein expressions, and downregulated Bax and caspase-3, which have been a major cause of cell death [48]. In this study, roscovitine also established an increased activity of Fak, Akt and GSK3β. Therefore, this implies that luteolin ameliorates apoptosis induced by MPP^+^ in SH-SY5Y cells through inhibiting the Cdk5-dependent Fak/Akt/GSK3β pathway.

## 4. Conclusions

In the present study, the protective efficacy of luteolin on MPP^+^-induced apoptosis was evaluated in SH-SY5Y cells. These findings indicate that luteolin protects MPP^+^-induced neuronal cell apoptosis. The inhibition of mitochondrial ROS-dependent oxidative stress and apoptosis were the major action of luteolin. Furthermore, luteolin also prevented Cdk5/p35 hyperactivity, resulting in enhancing Erk1/2/Drp1 and Fak/Akt/GSK3β signaling pathways in response to abolish MPP^+^-induced apoptosis, proposing a mechanism of luteolin on MPP^+^-induced cell apoptosis in SH-SY5Y cells. In conclusion, data in this present study provided clear evidence that luteolin offers neuroprotective against MPP^+^-induced apoptosis in SH-SY5Y, possibly by improving survival pathways. Luteolin could then represent a potential preventive agent for neuronal disorders.

## 5. Materials and Methods

### 5.1. Cell Culture

The human neuroblastoma SH-SY5Y cells were obtained from ATCC (Bethesda, Baltimore, MD, USA). The cells were maintained in DMEM supplemented with 10% *v/v* fetal bovine serum (FBS), 100 U/mL penicillin/streptomycin and 1% L-alanyl-L-glutamine. The cells were cultured in a condition of 5% CO_2_ and 95% humidified incubator at 37 °C.

### 5.2. Cell Viability Assay

SH-SY5Y cells were seeded at a density of 1 × 10^4^ cells/well onto a 96-well plate. The cells were treated with 1–100 µM MPP^+^ for 3, 9, 18 and 24 h or 2.5–80 µM luteolin for 24 h. For pre-treatment, the cells were pre-treated with 2.5–20 µM luteolin for 1 h, followed by 100 µM MPP^+^ for 24 h. After treatment, the cells were incubated with 0.5 mg/mL of 3-(4,5-dimethylthiazol-2-yl)-2,5-diphenyl tetrazolium bromide (MTT) at 37 °C for 4 h. Then, cultured mediums were removed, and the cells were dissolved in 100 µL DMSO. The activity was measured using absorbance at 570 nm with a microplate reader (Biochrom, Cambridge, UK). Results were expressed as percentage in comparison with control values.

### 5.3. Hoechst 33342 Straining

Apoptosis was examined by Hoechst 33342 staining. SH-SY5Y cells were seeded at a density of 1 × 10^6^ cells/dish onto a 35-mm dish for 24 h. After being treated with 100 µM MPP^+^ for 24 h with or without 20 µM luteolin pre-treatment for 1 h, the cells were washed with phosphate-buffered saline (PBS) and then stained with Hoechst 33342 (5 µg/mL) for 20 min at room temperature in the dark. The nuclei were visualized under a fluorescent microscope (Olympus, Tokyo, Japan).

### 5.4. Annexin V/7-ADD Staining 

The Annexin V FITC apoptotic detection kit was applied for apoptotic cells detection according to the supplier’s instructions. SH-SY5Y cells were seeded at a density of 1 × 10^6^ cells/dish onto a 35-mm dish for 24 h. The cells were pre-incubated with 20 µM luteolin for 1 h. After treatment with 100 µM MPP^+^ for 24 h, the cells were stained with 100 µL Annexin V/7-ADD solution, and incubated at room temperature for 20 min in the dark. The number of apoptotic cells was analyzed using a flow cytometry (Millipore, Burlington, MA, USA).

### 5.5. Intracellular ROS Measurement

The production of superoxide anion (O_2_^−^), hydrogen peroxide (H_2_O_2_) and hydroxyl radical (OH^−^) were determined by emitted fluorescence intensity measuring cell-permeable fluorescent dyes including DHE, DCF and HPF, respectively. SH-SY5Y cells were seeded at a density of 1 × 10^5^ cells/well onto a 24-well plate for 24 h. The cells were incubated with 10 µM DHE, DCF and HPF in the presence of 20 µM luteolin and 100 µM MPP^+^ for 1 h at 4 °C. Afterwards, the cells were incubated for 3 h at 37 °C. The intensity of fluorescence dyes was measured by flow cytometer (Merck, Darmstadt, Germany). Relative fluorescence was calculated as a ratio of the treated to the control fluorescence intensity.

### 5.6. Superoxide Anion (O_2_^−^) Production Assay

SH-SY5Y cells were seeded at a density of 1 × 10^6^ cells/dish onto a 35-mm dish for 24 h. The cells were pre-treated with 20 µM luteolin and 10 mM NAC for 1 h. The cells were then exposed to MPP^+^ at a concentration of 100 µM for 3 h. After the treatment, the cells were homogenated in a lysis buffer, and incubated in the solution (0.3 mM EDTA, 0.6 mM NBT, 0.1 mM xanthine and 20 µL xanthine oxidase) at 37 °C for 10 min. The absorbance was determined at 560 nm using a microplate reader (Biochrom, Cambridge, UK). Results were expressed as a percentage, in comparison with control values.

### 5.7. Lipid Peroxidation Measurement

Lipid peroxidation in SH-SY5Y cells was determined by the level of MDA. The cells were seeded at a density of 1 × 10^6^ cells/dish onto a 35-mm dish for 24 h. The cells were pre-treated with 20 µM luteolin and 10 mM NAC for 1 h. After 100 µM MPP^+^ treatment for 24 h, the cells were lysed and centrifuged at 14,000 rpm at 4 °C for 15 min. The supernatants were used to determine the MDA level by a thiobarbituric acid reactive substances (TBARS) assay. SDS (8.1%) containing acetic acid (20%) and TBA (0.8%) was added to each culture tube. Then, the samples were boiled at 90 °C for 60 min. After cooling, the samples were incubated with *n-butanol* and pyrimidine. The absorbance was determined at 532 nm using a microplate reader (Biochrom, Cambridge, UK). The level of TBARS was expressed as nM MDA/µg protein.

### 5.8. Measurement of Mitochondrial Membrane Potential (Δψm)

Δψm was detected using JC-1 dye. SH-SY5Y cells were seeded at a density of 1 × 10^6^ cells/dish onto a 35-mm dish for 24 h. The cells were incubated with 100 µM MPP^+^ for 0, 1,3, 6, 9, 12 and 24 h. For pre-treatment, the cells with pre-treatment of 20 µM luteolin for 1 h were given 100 µM MPP^+^ for 12 h. After completed experimental condition, the cells were then incubated with JC-1 dye for 30 min at 37 °C. Cells with low Δψm were measured using a flow cytometer (Millipore, Burlington, MA, USA).

### 5.9. Western Blotting Analysis 

SH-SY5Y cells were seeded at a density of 1 × 10^6^ cells/dish onto a 35-mm dish for 24 h. The cells were pre-treated with 20 µM luteolin and 10 µM roscovitine for 1 h, and then exposed with 100 µM MPP^+^ for 24 h. After the treatment, the cells were lysed in lysis buffer containing a freshly added protease inhibitor cocktail. Lysate was centrifuged at 14,000 rpm at 4 °C for 15 min. The supernatant was collected, and the concentration of total protein was measured using the Bradford assay (Bio-rad Laboratories, Hercules, CA, USA). Equal amounts of proteins (30 µg) in lysates were analyzed by SDS-PAGE and transferred onto PVDF membranes. Non-specific bindings were blocked with 5% skimmed milk or 3% BSA. The membranes were then incubated overnight with primary antibodies against alpha synuclein (α-synuclein), caspase-3, Bax, Bcl-2, Cdk5, GAP43 (Santa Cruz Biotechnology, Santa Cruz, CA, USA), Akt, p-Akt, cleaved caspase-3, cytochrome *c*, Drp1, p-Drp1, GSK3β, p-GSK3β, synapsin-1 (Cell Signaling Technology, Danvers, MA, USA), Erk1/2, p-Erk1/2, Fak, p-Fak, PSD95 (Abcam, Cambridge, UK) and tyrosine hydroxylase (TH) (Millipore, Billerica, MA, USA), followed by 2 h of incubation with a secondary antibody conjugated to HRP (Abcam, Cambridge, UK). Interested protein signals were detected using SuperSignal West Pico chemiluminescence substrate (Thermo Fisher Scientific, Rockford, IL, USA), and visualized by film exposion. The band density was quantified using ImageJ software (Java 8) using β-actin (Cell Signaling Technology, Danvers, MA, USA) for normalization. 

### 5.10. Statistical Analysis

Experimental data were presented as mean ± standard deviation (SD) based on three replicated experiments. One-way *ANOVA*, followed by Tukey’s *post-hoc*, was used to determine difference between multiple groups. A *p*-value of <0.05 was considered as a statistically significant difference in all tests. 

## Data Availability

Not applicable.

## Sample Availability

Samples of the compounds are not available from the authors.

## References

[1] Xu L., Pu J. (2016). Alpha-synuclein in Parkinson’s disease: From pathogenetic dysfunction to potential clinical application. Parkinsons Dis..

[2] Zhai S., Tanimura A., Graves S.M., Shen W., Surmeier D.J. (2018). Striatal synapses, circuits and Parkinson’s disease. Curr. Opin. Neurobiol..

[3] Kalia L.V., Kalia S.K., Lang A.E. (2015). Disease-modifying strategies for Parkinson’s disease. Movt. Disord..

[4] Cui W., Zhang Z., Li W., Hu S., Mak S., Zhang H., Han R., Yuan S., Li S., Sa F. (2013). The anti-cancer agent SU4312 unexpectedly protects against MPP(+)-induced neurotoxicity via selective and direct inhibition of neuronal NOS. Br. J. Pharmacol..

[5] Subramaniam S.R., Chesselet M.F. (2013). Mitochondrial dysfunction and oxidative stress in Parkinson’s disease. Prog. Neurobiol..

[6] Bhat A.H., Dar K.B., Anees S., Zargar M.A., Masood A., Sofi M.A., Ganie S.A. (2015). Oxidative stress, mitochondrial dysfunction and neurodegenerative diseases: A mechanistic. Biomed. Pharmacother..

[7] Song Z., Tao A. (2020). The neuroprotective effects of astragaloside IV against H_2_O_2_-induced damage in SH-SY5Y cells are associated with synaptic plasticity. J. Chem..

[8] Santos N.A.G., Martins N.M., Sisti F.M., Fernandes L.S., Ferreira R.S., Queiroz R.H.C., Santos A.C. (2015). The neuroprotection of cannabidiol against MPP+-induced toxicity in PC12 cells involves trkA receptors, upregulation of axonal and synapticproteins, neuritogenesis, and might be relevant to Parkinson’s disease. Toxicol. In Vitro.

[9] Zhou M., Xu S., Mi J., Ueda K., Chan P. (2013). Nuclear translocation of alpha-synuclein 742 increases susceptibility of MES23.5 cells to oxidative stress. Brain Res..

[10] Shah K., Rossie S. (2018). Tale of the good and the bad Cdk5: Remodeling of the actin 724 cytoskeleton in the brain. Mol. Neurobiol..

[11] Zhu J., Li W., Mao Z. (2011). Cdk5: Mediator of neuronal development, death and the response to DNA damage. Mech. Ageing Dev..

[12] Muangsab J., Prommeenate P., Chetsawang B., Chonpathompikunlert P., Sukketsiri W., Hutamekalin P. (2019). Protective effect of valproic acid on MPP+-induced neurotoxicity in dopaminergic SH-SY5Y cells through Cdk5/p35/Erk signaling cascade. Trop. J. Pharmaceut. Res..

[13] Roskoski R. (2012). ERK1/2 MAP kinases: Structure, function, and regulation. Pharmacol. Res..

[14] Kashatus J.A., Nascimento A., Myers L.J., Sher A., Byrne F.L., Hoehn K.L., Counter C.M., Kashatus D.F. (2015). Erk2 Phosphorylation of Drp1 Promotes Mitochondrial Fission and MAPK-Driven Tumor Growth. Mol. Cell..

[15] Lee Y., Lee H.Y., Hanna R.A., Gustafsson A.B. (2011). Mitochondrial autophagy by Bnip3 involves Drp1-mediated mitochondrial fission and recruitment of Parkin in cardiac myocytes. Am. J. Physiol Heart Circ. Physiol..

[16] Duan C., Kuang L., Xiang X., Zhang J., Zhu Y., Wu Y., Yan Q., Liu L., Li T. (2020). Drp1 regulates mitochondrial dysfunction and dysregulated metabolism in ischemic injury via Clec16a-, BAX-, and GSH- pathways. Cell Death Dis..

[17] Ma J.T., Zhang X.Y., Cao R., Sun L., Jing W., Zhao J.Z., Zhang S.L., Huang L.T., Han C.B. (2019). Effects of dynamin-related protein 1 regulated mitochondrial dynamic changes on invasion and metastasis of lung cancer cells. J. Cancer.

[18] Santos A.R.C., Corredor R.G., Obeso B.A., Trakhtenberg E.F., Wang Y., Ponmattam J., Dvoriantchikova G., Ivanov D., Shestopalov V.I., Goldberg J.L. (2012). β1 integrin-focal adhesion kinase (FAK) signaling modulates retinal ganglion cell (RGC) survival. PLoS ONE.

[19] Liu Z., Cai H., Zhang P., Li H., Liu H., Li Z. (2012). Activation of ERK1/2 and PI3K/Akt by IGF-1 on GAP-43 expression in DRG neurons with excitotoxicity induced by glutamate in vitro. Cell Mol. Neurobiol..

[20] Kong D., Chen F., Sima N. (2015). Inhibition of focal adhesion kinase induces apoptosis in bladder cancer cells via Src and the phosphatidylinositol 3-kinase/Akt pathway. Exp. Ther. Med..

[21] Zhou Q., Guo X., Choksi R. (2017). Activation of Focal Adhesion Kinase and Src Mediates Acquired Sorafenib Resistance in A549 Human Lung Adenocarcinoma Xenografts. J. Pharmacol. Ther..

[22] Jacobs K.M., Bhave S.R., Ferraro D.J., Jaboin J.J., Hallahan D.E., Thotala D. (2012). GSK3β: A bifunctional role in cell death pathways. Int. J. Cell Biol..

[23] Mansuri M.L., Parihar P., Solanki I., Parihar M.S. (2014). Flavonoids in modulation of cell survival signalling pathways. Genes Nutr..

[24] Nabavi S.F., Braidy N., Gortzi O., Sobarzo-Sanchez E., Daglia M., Skalicka-WoĨniak K., Nabavi S.M. (2015). Luteolin as an anti-inflammatory and neuroprotective agent: A brief review. Brain Res. Bull..

[25] Lin P., Tian X.H., Yi Y.S., Jiang W.S., Zhou Y.J., Cheng W.J. (2015). Luteolin-induced protection of H2O2-induced apoptosis in PC12 cells and the associated pathway. Mol. Med. Rep..

[26] Chang H., Li C., Huo K., Wang Q., Lu L., Zhang Q., Wang Y., Wang W. (2016). Luteolin Prevents H2O2-Induced Apoptosis in H9C2 Cells through Modulating Akt-P53/Mdm2 Signaling Pathway. BioMed Res. Int..

[27] Zhou W.B., Miao Z.N., Zhang B., Long W., Zheng F.X., Kong J., Yu B. (2019). Luteolin induces hippocampal neurogenesis in the Ts65Dn mouse model of Down syndrome. Neural. Regen. Res..

[28] Gu J., Cheng X., Luo X., Yang X., Pang Y., Zhang X., Zhang Y., Liu Y. (2018). Luteolin ameliorates cognitive impairments by suppressing the expression of inflammatory cytokines and enhancing synapse associated proteins GAP-43 and SYN levels in streptozotocin-induced diabetic rats. Neurochem. Res..

[29] Limboonreung L., Tuchindab P., Chongthammakun S. (2020). Chrysoeriol mediates mitochondrial protection via PI3K/Akt pathway in MPP+ treated SH-SY5Y cells. Neurosci. Lett..

[30] Sanders L.H., Greenamyre J.T. (2013). Oxidative damage to macromolecules in human Parkinson disease and the rotenone model. Free Radic. Biol. Med..

[31] Zhong J., Yu H., Huang C., Zhong Q., Chen Y., Xie J., Zhou Z., Xu J., Wang H. (2018). Inhibition of phosphodiesterase 4 by FCPR16 protects SH-SY5Y cells against MPP+-induced decline of mitochondrial membrane potential and oxidative stress. Redox. Biol..

[32] Hu L.W., Yen J.H., Shen Y.T., Wu K.Y., Wu M.J. (2014). Luteolin modulates 6-hydroxydopamine-induced transcriptional changes of stress response pathways in PC12 cells. PLoS ONE.

[33] More S.V., Choi D.K. (2017). Atractylenolide-I protects human SH-SY5Y cells from 1-Methyl689 4-phenylpyridinium-induced apoptotic cell death. Int. J. Mol. Sci..

[34] Garcia C.C., Blair H.J., Seager M., Coulthard A., Tennant S., Buddles M., Curtis A., Goodship J.A. (2004). Identification of a mutation in synapsin I, a synaptic vesicle protein, in a family with epilepsy. J. Med. Genet..

[35] Hung C.C., Lin C.H., Chang H., Wang C.Y., Lin S.H., Hsu P.C., Sun Y.Y., Lin T.N., Shie F.S., Kao L.S. (2016). Astrocytic GAP43 induced by the TLR4/NF-κB/STAT3 axis attenuates astrogliosis-mediated microglial activation and neurotoxicity. J. Neurosci..

[36] Phan J.A., Stokholm K., Zareba-Paslawska J., Jakobsen S., Vang K., Gjedde A., Landau A.M., Romero-Ramos M. (2017). Early synaptic dysfunction induced by α-synuclein in a rat model of Parkinson’s disease. Sci. Rep..

[37] Tseng Y.T., Lin W.J., Chang W.H., Lo Y.C. (2019). The novel protective effects of loganin against 1-methyl-4-phenylpyridinium-induced neurotoxicity: Enhancement of neurotrophic signaling, activation of IGF-1R/GLP-1R, and inhibition of RhoA/ROCK pathway. Phytother. Res..

[38] Liu Y., Fu X., Lan N., Li S., Zhang J., Wang S., Li C., Shang Y., Huang T., Zhang L. (2014). Luteolin protects against high fat diet-induced cognitive deficits in obesity mice. Behav Brain Res..

[39] Allen C.L., Bayraktutan U. (2009). Oxidative stress and its role in the pathogenesis of ischemic stroke. Int. Stroke.

[40] Lee D.H., Kim C.S., Lee Y.J. (2011). Astaxanthin protects against MPTP/MPP+-induced mitochondrial dysfunction and ROS production in vivo and in vitro. Food Chem. Toxicol..

[41] Patil S.P., Jain P.D., Sancheti J.S., Ghumatkar P.J., Tambe R., Sathaye S. (2014). Neuroprotective and neurotrophic effects of Apigenin and Luteolin in MPTP induced parkinsonism in mice. Neuropharmacology.

[42] Sharma P., Sharma M., Amin N.D., Sihag R.K., Grant P., Ahn N., Kulkarni A.B., Pant H.C. (2002). Phosphorylation of MEK1 by cdk5/p35 down-regulates the mitogen-activated protein kinase pathway. J. Biol. Chem..

[43] Arnold B., Cassady S.J., VanLaar V.S.V., Berman S.B. (2011). Integrating multiple aspects of mitochondrial dynamics in neurons: Age-related differences and dynamic changes in a chronic rotenone model. Neurobiol. Dis..

[44] Xie Z., Sanada K., Samuels B.A., Shih H., Tsai L.H. (2003). Serine 732 phosphorylation of FAK by Cdk5 is important for microtubule organization, nuclear movement, and neuronal migration. Cell.

[45] Lim S.T., Chen X.L., Lim Y., Hanson D.A., Vo T.T., Howerton K., Larocque N., Fisher S.J., Schlaepfer D.D., Ilic D. (2008). Nuclear FAK promotes cell proliferation and survival through FERM-enhanced p53 degradation. Mol. Cell.

[46] Jope R.S., Roh M.S. (2006). Glycogen synthase kinase-3 (GSK3) in psychiatric diseases and therapeutic interventions. Curr. Drug Targets.

[47] Lv J., Bai R., Wang L., Gao J., Zhang H. (2018). Artesunate may inhibit liver fibrosis via the FAK/Akt/β-catenin pathway in LX-2 cells. BMC Pharmacol. Toxicol..

[48] Jing Z., Wang C., Yang Q., Wei X., Jin Y., Meng Q., Liu Q., Liu Z., Ma X., Liu K. (2019). Luteolin attenuates glucocorticoid-induced osteoporosis by regulating ERK/Lrp-5/GSK-3β signaling pathway in vivo and in vitro. J. Cell Physiol..

